# Cost-effectiveness of guideline-based stepped and collaborative care versus treatment as usual for patients with depression – a cluster-randomized trial

**DOI:** 10.1186/s12888-020-02829-0

**Published:** 2020-08-28

**Authors:** Christian Brettschneider, Daniela Heddaeus, Maya Steinmann, Martin Härter, Birgit Watzke, Hans-Helmut König

**Affiliations:** 1 Department of Health Economics and Health Services Research, Hamburg Center for Health Economics, University Medical Center Hamburg-Eppendorf, Martinistraße 52, D-20251 Hamburg, Germany; 2 Department of Medical Psychology, University Medical Center Hamburg-Eppendorf, Martinistraße 52, D-20251 Hamburg, Germany; 3 Institute of Psychology, Clinical Psychology and Psychotherapy Research, University of Zurich, Binzmühlestrasse 14, Box 16, CH-8050 Zürich, Switzerland

**Keywords:** Depressive disorder, Costs and cost analysis, Quality-adjusted life years, Delivery of healthcare, integrated

## Abstract

**Background:**

Depression is associated with major patient burden. Its treatment requires complex and collaborative approaches. A stepped care model based on the German National Clinical Practice Guideline “Unipolar Depression” has been shown to be effective. In this study we assess the cost-effectiveness of this guideline based stepped care model versus treatment as usual in depression.

**Methods:**

This prospective cluster-randomized controlled trial included 737 depressive adult patients. Primary care practices were randomized to an intervention (IG) or a control group (CG). The intervention consisted of a four-level stepped care model. The CG received treatment as usual. A cost-utility analysis from the societal perspective with a time horizon of 12 months was performed. We used quality-adjusted life years (QALY) based on the EQ-5D-3L as effect measure. Resource utilization was assessed by patient questionnaires. Missing values were imputed by ‘multiple imputation using chained equations’ based on predictive mean matching. We calculated adjusted group differences in costs and effects as well as incremental cost-effectiveness ratios. To describe the statistical and decision uncertainty cost-effectiveness acceptability curves were constructed based on net-benefit regressions with bootstrapped standard errors (1000 replications). The complete sample and subgroups based on depression severity were considered.

**Results:**

We found no statically significant differences in costs and effects between IG and CG. The incremental total societal costs (+€5016; 95%-CI: [−€259;€10,290) and effects (+ 0.008 QALY; 95%-CI: [− 0.030; 0.046]) were higher in the IG in comparison to the CG. Significantly higher costs were found in the IG for outpatient physician services and psychiatrist services in comparison to the CG. Significantly higher total costs and productivity losses in the IG in comparison to the CG were found in the group with severe depression. Incremental cost-effectiveness ratios for the IG in comparison to the CG were unfavourable (complete sample: €627.000/QALY gained; mild depression: dominated; moderately severe depression: €645.154/QALY gained; severe depression: €2082,714/QALY gained) and the probability of cost-effectiveness of the intervention was low, except for the group with moderate depression (ICER: dominance; 70% for willingness-to-pay threshold of €50,000/QALY gained).

**Conclusions:**

We found no evidence for cost-effectiveness of the intervention in comparison to treatment as usual.

**Trial registration:**

NCT, NCT01731717. Registered 22 November 2012 - Retrospectively registered.

## Background

Depression affects society in different ways. The prevalence of depression is high [1], diagnosis is made and treatment initiated with a major delay [2–4], it is associated with a substantial disease burden in terms of loss of quality of life [5], worsens the course and prognosis of somatic diseases [6–8] and causes a high economic burden [9]. These challenges have been addressed by the development of systematic care approaches. In Germany, the National Clinical Practice Guideline “Unipolar Depression” [10, 11] recommends a stepped care approach based on collaborative principles [10]. The aim of stepped care is the supply of treatment at the least necessary intensity while constantly monitoring the course of disease [12].

Programs based on the stepped care approach have already been implemented and evaluated in different contexts. Systematic reviews conclude that stepped care could be at least as effective as usual care [13]. However, further need for research, e.g. into the specific characteristics of stepped services, the preferred model of delivery or the implementation of stepped care programs, is identified by these reviews [13, 14]. Considering the cost-effectiveness, there is evidence from several studies. However, most of these studies took a rather specific focus on stepped care approaches by evaluating the inclusion of digital measures into stepped care models [15–18] or by investigating stepped care in populations with specific underlying diseases [19–21] or in combination with other interventions [22, 23] or in specific populations [24–30]. There are three studies that show a certain degree of comparability to our study by evaluating stepped care exclusively for depression in a primary care sample. The study by Simon et al. is the least comparable study of those [31]. The authors of this study, who found that stepped care leads to substantially improved health with moderately increased costs, included only patients with depression persistent after 6–8 weeks of antidepressant treatment. This definition excludes huge numbers of patients and limits comparability to studies with broader inclusion criteria. A broader definition for inclusion was employed by Yan et al., who evaluated a stepped care treatment program compared to different usual care approaches. They found no clinical differences in health outcomes between the comparison groups [32]. However, they also identified potential cost savings. The study with the highest degree of comparability is the study by Meeuwissen et al., who assessed a stepped care programme based on a national treatment guideline [33]. They conducted a model-based economic evaluation of a stepped care program based on the Dutch guidelines. They found that this program is cost-effective compared to usual care.

To assess the effectiveness and cost-effectiveness of the German National Clinical Practice Guideline we transferred its recommendations into a program for clinical practice. The results of the effectiveness assessment have already been published [34]. The intervention, a guideline-based stepped care model (SCM), showed significantly higher odds of remission and response as well as a significant reduction of depression severity in comparison to the CG which received treatment as usual (TAU) [34]. However, the effectiveness assessment did not include the economic consequences of the intervention. While the assessment of effectiveness takes the benefit for patients into account, the assessment of economic consequences considers a wider perspective and provides evidence on the societal benefits by putting the health benefits into context to the costs caused by achieving these benefits. This supports policy makers in making informed decisions on the allocation of scarce healthcare resources. To provide this evidence, we performed a cost-effectiveness analysis comparing SCM and TAU in patients with depression over the course of 1 year from the perspective of the German society.

## Methods

### Sample

The details of this study (ClinicalTrials.gov: NCT01731717) have been reported elsewhere [35]. In summary, this analysis is based on a prospective cluster-randomized controlled trial. The intervention group (IG) was treated in the SCM. The control group (CG) received TAU. The treatments are described in detail below. Patient recruitment and inclusion was performed between August 2012 and March 2014 in 49 (IG: 36; CG: 13) primary care practices in Hamburg, Germany (Follow-ups: between 2012 and April 2015). The randomization process was not blinded and took place on the practice level. Randomization was performed by a computer program (minimisation based on location and size of practices and the income level of the district the practice is located in). The randomization scheme between IG and CG was 3:1. Patients were included if they had a score ≥ 5 on the Patient Health Questionnaire- (PHQ-) 9 (indicating a mild depression at minimum), were 18 years or older and gave informed consent. Patients were excluded if they had insufficient German language skills or if a disease or disorder made it impossible to complete the questionnaire. Additionally, patients were excluded if their main treatment focus was on a comorbid mental disorder and not on depression.

### Interventions

#### IG (SCM)

Patients in the IG received services from a stratified stepped and collaborative care program, including GP, psychiatrists, psychotherapists and psychiatric inpatient facilities. The intervention consisted of four steps. Step 1 incorporated active monitoring, Step 2 bibliotherapy, internet-based self-management and telephone-administered psychotherapy. Step 3 consisted of outpatient psychotherapy or antidepressant pharmacotherapy. In Step 4, a combination of psycho- and pharmacotherapy in an out- or inpatient setting was performed. The GP allocated the different interventions according to the guideline recommendations considering depression severity and patient preferences (shared decision making). For the initial depression treatment, patients received a specified depression diagnosis based on the ICD-10 criteria as recommended in the National Clinical Practice Guideline. This included information on subtype and disease severity. Monitoring and treatment adaption was performed based on the assessment of the PHQ-9 in regular intervals. A stepping up of treatment intensity was recommended in case that depression severity had not improved by at least 20% since the last contact. Additionally, an online platform displaying vacant treatment capacities in secondary care, a provider network, intensive training of GP regarding guideline recommendations and quarter-yearly quality circles were introduced.

#### CG (tau)

A diagnosis based on the ICD-10 criteria was not determined for patients in the CG. These patients were able to receive every approved treatment. This includes outpatient as well as inpatient psychotherapeutic or psychiatric services. GP in the CG had no access to the online platform, the provider network, the training regarding guideline recommendations or the quarter-yearly quality circles.

### Data collection and measures

#### Data collection

Data were collected at four time points by means of self-reported questionnaires which were returned by mail: baseline (T0), after 3 months (T1), after 6 months (T2) and after 12 months (T3). Accordingly, the time horizon of the study was 1 year.

We assessed sociodemographic information, type of health insurance, employment status, social support (F-SOZU-14 [36]), the symptom severity of depression (PHQ-9 [37, 38]) and the physical and mental health status (Physical Component Score (PCS) and Mental Component Score (MCS) of the Short-Form-12 (SF-12) [39–41]). Main outcomes of the cost-effectiveness analysis were quality-adjusted life years (QALY) in the 12-month period between T0 and T3 (EQ-5D-3L as measure of preference-based health-related quality of life (HRQL) [42]) and total 12-month costs calculated based on service utilization measured by a modified German version of the Client Sociodemographic and Service Receipt Inventory (CSSRI) [43].

#### Measurement of effects: EQ–5D-3L and QALY

The EQ–5D-3L consists of five domains measuring current problems in the dimensions: mobility; self-care; usual activities; pain/discomfort; and anxiety/depression [42]. There are three response levels for each domain: 1, no problems; 2, moderate problems; 3, extreme problems. Based on the patient’s response, it is possible to construct a utility score (EQ-5D index score). These utility scores represent preference-based valuations of HRQL derived from the general population. We used British [44], instead of German EQ-5D index scores [45] in this study as the German EQ-5D index scores are influenced by a major shortcoming. The available German TTO-based value set was derived in a rather small sample of the German general population (*n* = 334). This is likely to have led to a lack of statistical power in the regression model used to estimate the German value set. As a result, moderate or severe problems in the dimension usual activities and moderate problems in the dimension anxiety/depression are not associated with a decrement in the valuation of health states. This results in substantially higher EQ-5D index scores (total sample mean: 0.77 (SD: 0.24)) compared to the British value set that might not reflect societal preferences. Despite potential cultural differences in preferences for health states between the German and the British population, we believe that the British value set is more useful to value health states in our sample. Additionally, we want to point out that using the British value set in a non-UK-based study is a frequently implemented approach [46–49].

The EQ-5D has been validated in populations with depression [49, 50].

QALY were calculated separately for each period between time points. These values were summed up to gain 12-month QALY. The calculation was based on the assumption that the development of quality of life between two time points follows a linear trend. This means that the EQ-5D indices of two following time point, e.g. T2 and T3, were added and afterwards divided by 2 to gain the mean HRQL for this period. This mean HRQL value was multiplied with the observation time of the specific patient to calculate the QALY.

#### Questionnaire of service utilization

As there is no official standard for economic evaluations to inform decision-making in Germany, we adopted the societal perspective to assess the various effects of the intervention on healthcare delivery, family support and productivity. In contrast to the assessment of the other instruments, we measured service utilization at T0, T2 and T3, not at T1. The questionnaires asked the participants to recall their service utilization in the preceding 6 months. We considered inpatient services (general hospitals, psychiatric clinics, and rehabilitation clinics), outpatient physician services (GP + 21 specialists), outpatient non-physician services (e.g. physiotherapy, occupational therapy, and exercise therapy), outpatient psychotherapist services, medication, ambulatory nursing care and informal care. Additionally, productivity losses due to sick leave and treatment appointments (absenteeism) were assessed. Resource utilization of services in Step 2 were extracted from the study documentation.

#### Unit costs

Costs were calculated in Euro at the price level of 2012, the year the study started. As the time horizon of the study was 1 year, costs were not discounted.

Detailed information regarding the unit costs is shown in Table 1. German standardised unit costs developed by Bock et al. [51] were used for all categories, except for medication. The monetary valuation of medication was based on drug codes, dosage and duration and was valued based on the `Rote Liste´, a German pharmaceutical database [52]. Costs for inpatient services were calculated on a per day base by hospital type. Outpatient physician services and outpatient psychotherapist services were valued by means of average costs per contact. Outpatient non-physician services were calculated based on reimbursement schemes of the German statutory sickness funds per contact. Ambulatory nursing care assessed in hours was valued using the reimbursement schemes of the German statutory sickness funds. Informal care was valued using the replacement cost method assuming that a professional caregiver could have substituted informal care. Thus, the duration of informal care was valued using the hourly wage rate of workers in the commercial sector `Social care for older adults and disabled persons´ [51]. Productivity losses were valued based on the human capital approach by using mean gross income plus nonwage labour costs [53].

**Table 1:** Cost categories and sources of applied unit costs

Sector	Service / Goods	Units	Monetary values (unit costs)
Inpatient services	General hospitals, psychiatric hospitals and hospitals for rehabilitation	Days	Type specific mean rates [51]
Outpatient physician services	GP, specialists (e.g. cardiologist, internist, ophthalmologist)	Contacts	Type specific mean rates [51]
Outpatient non-physician services	e.g. physiotherapy, massage, lymph drainage, ergotherapy	Contacts	Reimbursement schedule [51]
Outpatient psychotherapist services	Psychotherapist	Contacts	Reimbursement schedule [51]
Medication	Product	Quantity	Official pharmaceutical index (Rote Liste) [52]
Nursing care	Ambulatory nursing care	Hours	Type specific wage [51]
	Informal care	Hours	Type specific wage (replacement cost approach) [51]
Indirect costs	Productivity losses	Hours	Gross income plus nonwage labor costs [53]

#### Intervention costs

Intervention costs were calculated for Step 2 services only. In steps 1, 3 and 4, outpatient physician or psychotherapeutic services, drug prescriptions and inpatient services were delivered. These costs were assessed and presented in the specific categories mentioned above.

As the intervention in step 2 consists of three services (bibliotherapy, internet-based self-management, telephone-administered psychotherapy), intervention costs represent the sum of costs caused by these three services. Bibliotherapy was valued by the price of the book (€15). Internet-based self-management was priced by the license fee of the self-management program (€250). Usually, the validity of the license is limited to 6 months. If a participant used the program between baseline and T2 as well between T2 and T3, we assumed that he or she required two licenses. Costs for telephone-administered psychotherapy were calculate by the product of the number of contacts and a price of €40 per contact. This corresponds to the wage paid to the psychotherapist per session.

### Statistical analysis

Analyses were performed based on the complete sample (base case analysis) as well as for subgroups of patients with different depression severity. As we had information on the specific ICD-10 diagnosis only in the IG, but information on the baseline values of the PHQ-9 in IG and CG, subgroups in the IG and the CG were defined by the baseline values of the PHQ-9. According to cut-off values extracted from the literature [54], a score of 5–9 constituted mild depression, a score of 10–14 moderate depression, a score of 15–19 moderately severe depression and a score of 20–27 severe depression. The subgroup analysis based on severity was defined a priori in the study protocol [35].

All analyses were performed with STATA 15 (StataCorp, College Station, USA). Results were considered statistically significant at *p* ≤ .05.

#### Imputation of missing values

Missing values were imputed on item level by ‘multiple imputation using chained equations’ (MICE) by fully conditional specification and based on predictive mean matching [55–58]. We used sociodemographic characteristics, comorbidities, disease-specific measures, and health care utilisation as covariates in the imputation models (in total: 236 variables either with or without missing values). The proportion of missing values at baseline ranged from 0% (Age) and 27% (Number of hours absent from work due to physician appointments). 48% of the participants (IG: 48%; CG: 49%) had no missing values. Loss to follow-up was 39% (IG: 40%; CG: 36%).

The imputation was based on sociodemographic, clinical and economic data assessed at baseline as well at T2 and T3 and was performed under fully conditional specification [58, 59]. Regarding the number of imputations, we decided to follow the suggestions made by van Buuren [59] and based the number of imputed datasets on the percentage of missing values in the variable with the most missing values at baseline (Numbers of hours absent from work due to physician appointments: 27%). Therefore, the following analyses are based on 30 datasets with *N* = 737 participants per data set (IG: 569; CG: 168). The results based on each of the imputed datasets were pooled by applying Rubin’s rules [57].

#### Comparison of baseline characteristics

We used linear and logistic mixed-effects regression models to identify baseline differences between IG and CG. The analyses were unadjusted considering only the treatment group as independent variable and the primary practice as random effect.

#### Comparison of total costs, cost categories and effects after 12 months

The analyses in the complete sample and the subgroups were adjusted for baseline variables with differences at a *p*-value of 0.1. This implies:

Complete sample: Age, employment status, PCS, MCS.

Mild depression: Age, social support.

Moderate depression: Age, employment status, PCS, MCS, social support, baseline HRQL.

Moderately severe depression: Type of health insurance, depression severity.

Severe depression: baseline HRQL.

Additionally, we considered the specific baseline costs in all analytical models.

We constructed linear mixed models with the aforementioned covariates as fixed effects and the primary care practice as random effect. To address the issue of the skewness of cost data, we calculated bootstrapped standard errors based on 1000 replications. This number of replications was frequently applied in recent economic evaluations [60–63].

#### Calculation of the ICER as point estimate of cost-effectiveness

We calculated the incremental cost effectiveness ratio (ICER) as a point estimate of cost-effectiveness. The ICER is a ratio and consists of the differences between IG and CG in mean total costs ($\overline{C}$) in the numerator and mean effects ($\overline{E}$) in the denominator:

$$ ICER=\frac{{\overline{C}}_{IG}-{\overline{C}}_{CG}}{{\overline{E}}_{IG}-{\overline{E}}_{CG}}=\frac{\Delta  \overline{C}}{\Delta  \overline{E}} $$

As there is no official German threshold to consider an ICER cost-effective, we applied the widely used threshold of €50,000/QALY gained [64].

#### Calculation of the CEAC as assessment of uncertainty

As the ICER is a point estimate considering only mean values of costs and effects, it provides no information on the uncertainty in the analysis. For this reason, we constructed cost-effectiveness acceptability curves (CEAC) based on a series of net-benefit regressions [65, 66].

In a first step, the patient-specific net benefit (NB) *NB*_*i*_ = *E*_*i*_ × *λ* − *C*_*i*_ was calculated. The NB consists of the individual 12-month costs in € (C_i_), the individual 12-month effect in QALY (E_i_) and a willingness-to-pay (WTP) margin in €/QALY gained (λ). To construct a CEAC, the individual NB is used as dependent variable in a regression model, while group is used as independent variable. This procedure is repeated for different WTP margins. In case of our study, we used WTP margins ranging from €0/QALY gained to €130.000/QALY gained and proceeded in ‘*€10.000/QALY gained’* steps. To present the CEAC graphically, the different WTP margins are plotted on the x-axis and the probabilities of cost-effectiveness are plotted on the y-axis. The probability of cost-effectiveness at a WTP margin corresponds to the 0.5 x the *p*-value of the coefficient of the group difference in the net-benefit regressions in case the coefficient is negative and 1–0.5 x the p-value if the coefficient is positive. For a rationale of this approach, please see Hoch et al. [65].

We used the same regression approach and adjusted for the same covariates as for the comparison of costs and effects (step 3).

## Results

### Characteristics of the study population at baseline

The mean age of the population was 42.9 years (SD: 14.0; Range: 18–88), the majority was female (73%). The percentage of participants living with a partner was 59%. The mean symptom severity of depression was moderately severe (mean PHQ-9: 15.0; SD: 4.8). The PHQ-9 identified 93 patients as mildly, 232 as moderately 271 patients as moderately severe and 141 from as severely depressed. Mean HRQL (EQ-5D index) was 0.57 (SD: 0.27). Patients in the IG were more frequently employed than patients in the CG (IG: 78%; CG: 69%; *p* < .05). No other differences reached statistical significance at a level of *p* ≤ .05 (Table 2).

**Table 2:** Sociodemographic characteristics of the complete sample at baseline

Characteristic	Intervention group (*n* = 569)	Control group (*n* = 168)	*p*-value
**Age (years)**			
mean (SD)	42.09 (13.45)	45.60 (15.45)	0.07
**Female: %**	72.41	76.19	0.38
**Single: %**	59.03	56.83	0.64
**Private HI: %**	5.55	8.73	0.25
**Employed: %**	77.56	68.53	0.04
**Severity of depression (PHQ-9)**			
mean (SD)	15.29 (4.68)	14.09 (4.91)	0.17
**Total costs (€)**			
mean (SD)	5636 (8297)	7688 (10,764)	0.10
**EQ-5D Index**			
mean (SD)	0.58 (0.26)	0.53 (0.28)	0.22
**Physical Health Status (SF-12)**			
mean (SD)	44.48 (10.52)	42.15 (10.31)	0.06
**Mental Health Status (SF-12)**			
mean (SD)	28.56 (8.41)	30.56	0.07

### Complete sample: costs and effects in IG and CG

We found that patients in the IG caused mean total costs of €23.920 (SD: €28.421), while mean total costs in the CG were €21.430 (SD: €23.506) (Table 3). The share of productivity losses in total costs was higher than that of costs for healthcare and for support by family in both groups (IG: 60%; CG: 56%). Healthcare costs cost were mainly caused by inpatient services (IG: 69%; CG: 70%). The number of QALY over the course of 12 month was 0.65 (SD: 0.23) in the IG and 0.61 (0.23) in the CG.

**Table 3:** Mean costs and mean effects per group in the complete sample and subgroups over the course of twelve months (€ 2012)

	Total sample (*n* = 737)		Subgroup: Mild depression (n = 93) (PHQ-9: 5–9)		Subgroup: Moderate depression (*n* = 232) (PHQ-9: 10–14)		Subgroup: Moderately severe depression (*n* = 271) (PHQ-9: 15–19)		Subgroup: Severe depression (*n* = 141) (PHQ-9: 20–27)	
	Intervention group (*n* = 569)	Control group (*n* = 168)	Intervention group (*n* = 63)	Control group (*n* = 30)	Intervention group (*n* = 175)	Control group (*n* = 57)	Intervention group (*n* = 221)	Control group (*n* = 50)	Intervention group (*n* = 110)	Control group (*n* = 31)
	Mean (SD)	Mean (SD)	Mean (SD)	Mean (SD)	Mean (SD)	Mean (SD)	Mean (SD)	Mean (SD)	Mean (SD)	Mean (SD)
Total costs (€)	23,920 (28,421)	21,430 (23,506)	20,228 (27,672)	13,798 (17,119)	17,729 (22,325)	21,443 (24,051)	24,647 (29,292)	24,400 (24,964)	34,425 (32,533)	23,999 (24,456)
Healthcare costs (€)	8955 (16,990)	8724 (14,569)	8576 (18,104)	5359 (8910)	6083 (13,080)	8513 (13,170)	9346 (17,920)	8859 (15,794)	12,953 (19,088)	12,149 (18,559)
Inpatient services (€)	6140 (15,713)	6129 (13,180)	5583 (15,738)	3507 (8362)	3612 (12,111)	6030 (11,936)	6361 (16,423)	6035 (14,805)	10,037 (18,413)	9002 (15,959)
Psychiatric hospital (€)	926 (4927)	1807 (6313)	1419 (5639)	542 (3845)	605 (5660)	1219 (5605)	837 (4338)	2531 (8794)	1332 (4575)	2943 (7847)
Outpatient physician services (€)	1867 (1591)	1571 (1543)	1542 (1553)	1022 (962)	1663 (1446)	1571 (1644)	2015 (1706)	1584 (1367)	2078 (1540)	2082 (1909)
Psychiatrist (€)	199 (372)	110 (250)	178 (347)	61 (192)	160 (346)	108 (260)	217 (394)	142 (272)	239 (376)	110 (236)
Psychotherapist (€)	950 (1174)	761 (1257)	638 (1028)	261 (601)	894 (1151)	793 (1398)	1025 (1222)	801 (1142)	1069 (1164)	1122 (1502)
Outpatient non-physician services (€)	240 (524)	267 (528)	248 (578)	248 (399)	153 (357)	294 (597)	289 (624)	231 (455)	274 (482)	291 (622)
Drugs (€)	463 (1993)	700 (2172)	887 (3721)	570 (1012)	417 (2024)	517 (781)	446 (1701)	956 (3375)	328 (547)	750 (2297)
Ambulatory care (€)	26 (210)	47 (300)	27 (197)	4 (71)	41 (290)	87 (464)	15 (154)	41 (198)	24 (157)	23 (155)
Intervention in step 2 (€)	196 (279)	0 (0)	253 (309)	0 (0)	179 (265)	0 (0)	197 (285)	0 (0)	188 (265)	0 (0)
Costs of familiy support										
Informal care (€)	623 (2952)	607 (2423)	627 (3182)	115 (1101)	257 (1700)	627 (1951)	844 (3569)	867 (3114)	760 (2978)	626 (2802)
Productivity losses (€)	14,342 (17,105)	12,099 (15,161)	11,026 (15,329)	8324 (12,141)	11,389 (14,567)	12,303 (15,551)	14,457 (16,708)	14,674 (17,023)	20,712 (20,609)	11,224 (13,383)
QALY (EQ-5D: UK-Tarif)	0.65 (0.23)	0.61 (0.23)	0.68 (0.21)	0.67 (0.24)	0.71 (0.18)	0,64 (0.19)	0.63 (0.24)	0.61 (0.23)	0.58	0,50 (0,26)

Regarding group differences, total costs as well as healthcare costs, cost for support by the family and productivity losses were higher in the IG than in the CG (Table 4). However, these differences did not achieve statistical significance. Significantly higher costs in the IG compared to the CG were found for outpatient physician services (mean: +€467; 95%-CI: [€126;€808]) and interventional services in step 2 (mean: +€218; [95%-CI: €196;€266]). Regarding the effects, the IG gained more QALY than the CG, although on a statistically non-significant level.

**Table 4:** Differences in costs and effects in the complete sample and per subgroup (€ 2012)

	Total sample (*n* = 737)		Subgroup: Mild depression (*n* = 93) (PHQ-9: 5–9)		Subgroup: Moderate depression (*n* = 232) (PHQ-9: 10–14)		Subgroup: Moderately severe depression (*n* = 271) (PHQ-9: 15–19)		Subgroup: Severe depression (*n* = 141) (PHQ-9: 20–27)	
	Mean (€)	95%-CI	Mean (€)	95%-CI	Mean (€)	SE (€)^#^	Mean (€)	SE (€)^#^	Mean (€)	SE (€)^#^
Total costs	5016	(−259; 10,290)	9731	(− 3017; 22,478)	−628	(−7442; 6186)	6047	(− 3084; 15,178)	14,579*	(2785; 26,373)
Healthcare costs	2712	(− 641; 6065)	5966	(− 2568; 14,500)	−23	(− 4671; 4625)	3321	(− 1666; 8308)	4324	(− 2982; 11,630)
Inpatient services	1769	(− 1346; 4884)	3468	(− 3589; 10,526)	− 453	(− 4889; 3982)	2487	(− 1737; 6712)	2911	(4202; 10,024)
Psychiatric hospital	−202	(− 1216; 811)	1101	(− 1649; 3851)	−660	(− 2334; 1015)	196	(− 1054; 1445)	− 1695	(− 5370; 1980)
Outpatient physician services	467*	(126; 808)	723*	(134; 1311)	347	(− 120; 815)	832*	(338; 1327)	266	(− 365; 896)
Psychiatrist	95*	(37; 153)	84	(−65; 234)	79	(−42; 201)	77	(−31; 185)	123	(−36; 281)
Psychotherapist	239	(−37; 515)	250	(− 147; 648)	244	(− 207; 696)	242	(−190; 674)	75	(−533; 683)
Outpatient non-physician services	37	(−59; 132)	46	(−192; 283)	−57	(−216; 102)	50	(−97; 197)	51	(−178; 281)
Drugs	−43	(−43; 308)	256	(− 565; 1077)	49	(− 304; 402)	−199	(− 894; 497)	−67	(− 371; 237)
Ambulatory care	−8	(−47; 30)	32	(−55; 120)	6	(− 123; 136)	−24	(− 87; 40)	124	(−279; 528)
Intervention in step 2	218*	(169; 266)	285*	(159; 411)	195*	(131; 258)	204*	(134; 275)	203*	(117; 290)
Costs of family support										
Informal care	82	(−567; 732)	543	(− 848; 1933)	−162	(− 1135; 811)	−72	(− 1092; 947)	434	(− 1583; 2450)
Productivity losses	2238	(− 668; 5145)	2754	(− 3438; 8945)	− 279	(− 4304; 3744)	1855	(− 4362; 8072)	10,646*	(3627; 17,666)
QALY (EQ-5D: UK-Tarif)	0.008	(−0.030; 0.046)	−0.010	(− 0.122; 0.101)	0.028	(− 0.044; 0.100)	0.013	(−0.058; 0.083)	0.007	(− 0.097; 0.111)

*p < .05;

### Subgroups: costs and effects in IG and CG

The total costs in the IG ranged from €17,729 (SD: €22,325) for patients with moderate depression to €34,245 (SD: €32,533) for those with severe depression (Table 3). In the CG the range was from €13,798 (SD: €17,119) in the group with mild depression to €24,400 (SD: €24,964) in the group with moderately-severe depression. In all subgroups productivity losses had a larger share in total costs (range: 55–64%) than costs for healthcare and for support by the family, except for the CG in the subgroup with severe depression (47%).

Comparing the development of costs in IG and CG in the subgroups, we found that mean healthcare costs in the CG increased with increasing depression severity. The healthcare costs in the subgroups of patients with moderate and moderately severe depression were comparable, with the exception of drugs. Regarding drugs, patients with moderately severe depression caused the highest mean costs of all subgroups.

In the IG, a comparable trend of increasing average healthcare costs with increasing disease severity can be observed for patient with moderate, moderately severe and severe depression. A special pattern can be observed in the subgroup of patients with mild depression. In comparison to patients with moderate depression, these patients caused higher mean healthcare costs (€8576 vs. €6083), mean costs for inpatient services (€5583 vs. €3612) and mean drug costs (€887 vs. €417). Of particular note is that the subgroup with mild depression caused the highest mean drug costs and mean costs for psychiatric inpatient services of all subgroups.

In the subgroups, patients in the IG with mild (mean: +€723; 95%-CI: [€134;€1311]); or moderately severe (mean: +€832; 95%-CI: [€338;€1327]) depression caused higher costs for outpatient physician services than those in the CG. The group of patients with moderate depression showed no significant cost differences. However, the total costs in the subgroup with moderate depression were lower in the IG compared to the CG, yet at a non-significant level (mean: -€628; 95%-CI: [−€7442;€6186]). In the group of patients with severe depression, total costs (mean: +€14,579; 95%-CI: €2785;€26,373]) and productivity losses (mean: +€10,646; 95%-CI: [€3627;€17,666]) were significantly higher in the IG than in the CG. The difference in productivity losses was mainly caused by significantly higher costs in the IG compared to the CG between baseline and T2, i.e. in the first 6 months (mean: +€7593; 95%-CI: [€2142;€13,044]). There were no significant QALY differences between IG and CG in the subgroups.

### Point estimates of cost-effectiveness

In the complete sample, the ICER was unfavourable (€627,000/QALY gained). In the group with mild depression, the IG was dominated by the CG which means that the IG caused higher costs but gained fewer QALY than the CG. In the group with moderate depression, the IG was dominant as costs were lower and effects were higher than in the CG. In the remaining groups there were unfavourable ICER of €465,154/QALY gained (moderately severe depression) and €2082,714/QALY gained (severe depression).

### Uncertainty analyses of cost-effectiveness

**Fig. 1:**
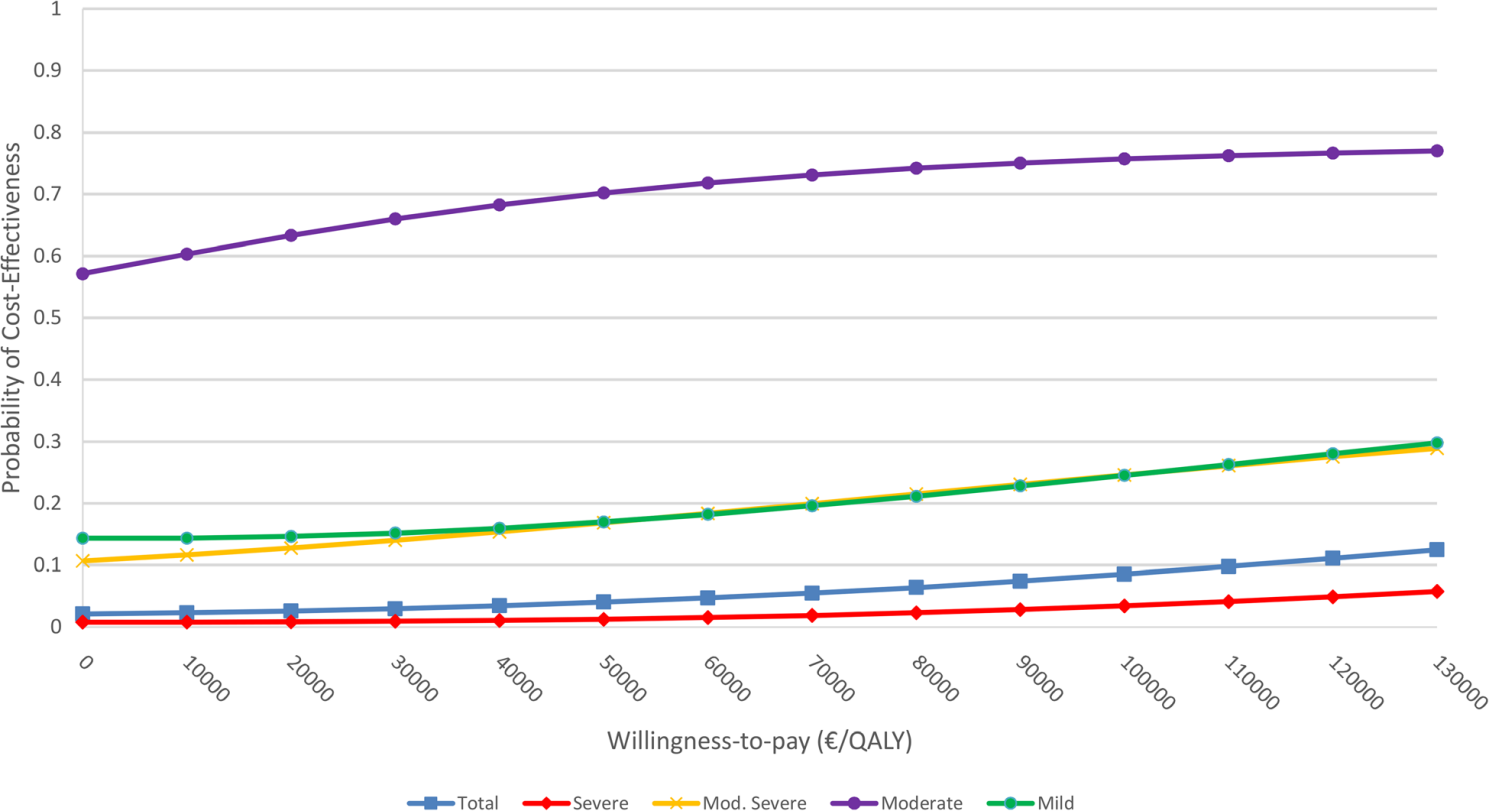
Cost-effectiveness acceptability curves for the complete sample and the subgroups by depression severity

Figure 1 shows the CEAC for the different groups. The CEACs show three different patterns.

Pattern one (complete sample and severe depression) shows a rather flat slope on a very low level of probability for cost-effectiveness. This indicates that the probability that IG is cost-effective compared to CG is low for all possible WTP values. Regarding the subgroup of patients with severe depression, the probability of cost-effectiveness of IG compared to CG was 2.5% at the WTP margin of €50.000/QALY gained. By implication, this means that the CG has a 97.5% probability of being cost-effective, which meets the margin of error of the statistical test and hence is an indicator that CG is cost-effective in the group of patients with severe depression. The second pattern (mild and moderately severe depression) shows also a rather low probability of cost-effectiveness of the intervention (between 10 and 30%). As a third pattern, the group with moderate depression shows an already elevated probability of 57% at the minimum WTP, which increases to 78%. Using the WTP margin of €50,000/QALY gained, the CEAC indicates a 70% probability of cost-effectiveness of the intervention.

## Discussion

Our analysis failed to provide sufficient evidence that the intervention in the IG is cost-effective. In case of severe depression, the evidence represented by the CEAC even indicates that treatment as usual is preferable from an economic point of view. As the conclusiveness of this statement might not be easily comprehensible for readers not familiar with the interpretation of the CEAC, we want to explain this. We constructed the CEAC by the NMB regression approach. As we wanted to indicate the probability of cost-effectiveness of the intervention in the IG, we coded TAU as 0 (reference group) and the intervention as 1. As we considered only two groups in this regression, the probability of TAU being cost-effective is the counter-probability of intervention in the IG being cost effective. Hence, if this probability is 2.5%, the probability of TAU being cost-effective is 97.5%. In our analyses the margin of error was set to α = .05. As the CEAC is a one-sided test a probability of ≥97.5% can be considered as conclusive. This means we can say that TAU is cost-effective in this subgroup. These results are not in line with the findings by Härter et al., who observed for patients in the IG a pronounced improvement of symptom burden as well as increased odds of response and remission [34]. Nevertheless, in the IG some indicators for an impact of the intervention on healthcare delivery can be identified.

There are two significant observation that suggest the existence of such effects. First, the National Clinical Practice Guideline recommends low intensity treatments for patients with mild depression [10]. In our study, these interventional measures (bibliotherapy, web-based self-management, telephone psychotherapy) showed the highest costs and incremental costs in this group of patients in comparison to other degrees of depression severity. Second, the National Clinical Practice Guideline lays a strong emphasis on treatment in the outpatient sector by mental health professionals [10]. In the complete sample, we found that the costs for psychiatric outpatient services were significantly higher in the IG than in the CG. The same trend was found for all subgroups and the psychotherapeutic services. This can be interpreted as in line with the National Clinical Practice Guideline [10]. Additionally, we found the same trend of increasing costs with increasing depression severity, at least for moderate, moderately severe and severe depression. For outpatient mental health services there were also higher costs in the IG compared to the CG in all three subgroups. The National Clinical Practice Guideline is built on the idea that patients should receive treatment at an intensity level that matches the demands caused by the disease [10]. Hence, even if we assume that the GP in the CG are aware of at least some recommendations of the National Clinical Practice Guideline and that this influences the increasing treatment intensity in the CG, the existence of the same trend and the, partially non-significant, higher costs for outpatient mental health services in the IG can cautiously be seen as an indicator for the influence of the improved knowledge of the National Clinical Practice Guideline and the intervention.

However, some results in the subgroup of mild depression deserve special attention. In the interpretation of these unexpected findings, we have to keep in mind that this subgroups was rather small (*n* = 93). The healthcare costs in this subgroup were much, yet not significantly, higher in the IG than in the CG. Apart from general hospital services, there were higher costs for mental health specific services (inpatient psychiatric, psychiatrist and psychotherapist services) as well as for drugs in the IG compared to the CG. The National Clinical Practice Guideline recommends for these patients watchful waiting and low threshold interventions, like those in step 2. As these services were often utilized in this group, the National Clinical Practice Guideline recommendations seem to have been effective. However, it might have been the case that GP in the IG by having better access to psychotherapist services (e.g. by the online platform for vacant therapy places) brought mild patients into treatment that they were not intended to receive based on the National Clinical Practice Guideline. That would mean, that we might have observed a disincentive in this group, which resulted in an overutilization of services. If this is the case we identified a misallocation that could be caused for example by an inefficient education or by altruistic acts.

Comparing our results to the results of previous studies is -as shown in the Background- limited by the diverse and partially even highly specific nature of these analyses. Even a comparison to the study conducted by Simon et al., who treated patients with depression in a primary care setting, is limited by the facts that the authors (a) only included patients with depression persistent after 6–8 weeks of antidepressant treatment and (b) used depression-free days at outcome measure [31]. This reduces the comparability of the results to a high extent. Hence, we only refer to the studies of Yan et al. and Meeuwissen at al [32, 33]. In these studies the stepped care approach was used for treatment of depression in general in the adult population in a primary care setting. Their results diverge from our results. Meeuwissen et al. concluded that stepped care was cost-effective at a high probability [33] while Yan et al. identified a potential for cost savings [32]. As highlighted in the review by van Straten et al., there are often differences in the characteristics of the stepped care approaches [14]. This could be an explanation for the differences in results between the study by Yan et al. and our study. Yan et al. evaluated a two-step program considering patients with a PHQ-9 score of 10 and higher and treated patients with moderate depression (PHQ-9 score: 10–14) by watchful waiting and self-management, and patients moderately severe or severe depression (PHQ-9 score: 15–27) with more intense treatments [32]. We evaluated a four-step program considering patients with a PHQ-9 score of 5 and higher, and treated patients with mild depression (PHQ-9 score: 5–9) by watchful waiting and low-intensity interventions, patients with a moderate depression (PHQ-9 score: 10–14) with outpatient pharmacotherapy or psychotherapy and patients with moderately severe or severe depression (PHQ-9 score: 15–27) with pharmaco- and psychotherapy in an outpatient or even inpatient setting. Hence, in comparison to Yan et al. we have treated patients already at a lower disease severity and treated them with more intensity at an earlier stage of disease. This means that our intervention had a higher intensity and could have caused extra costs in comparison to cost savings. The differences to Meeuwissen et al. might be explained by methodological differences. This group evaluated a stepped care approach based on the Dutch Multidisciplinary Guideline for Depression by conducting a model-based study based on a Dutch disease model [33]. We conducted a trial-based study situated in the catchment area of Hamburg, Germany. As the German healthcare system shows only a low level of service integration and is characterized by prolonged waiting periods for psychotherapy [67], our intervention, which is based on cooperation and swift adaption to new circumstances, needed to adapt the traditional service routines. Over the course of 1 year, the loss due to efforts of adaption might have been too large to be offset by gains of efficiency. As a model-based study is not faced with these issues of implementation, we might conclude that the study by Meeuwissen et al. [33] represents the cost-effectiveness of a well-established and fully integrated stepped care programme, while our trial-based study might be influenced by implementation effects.

Considering the aforementioned aspect of the study, we can identify the time horizon of 1 year as the first limitation of this study. Besides the implementation effects we might have assesses, long-term effects of the intervention were not observed. Due to the natural course of depression, the duration and number of episodes, the duration of remission and the risk of relapses, 1 year might be too short to observe all differences between the interventions [68, 69]. It is possible that the intervention by reducing the risk of relapses or duration of episodes might even has an impact on the reported negative effects of a high mental health burden on physical health [3, 70]. This could have an influence on the healthcare costs. The second potential limitation is the effect measure. Härter et al. showed that the intervention reduces symptom severity, leads to more remissions, and improves the physical health status (measure by the PCS of the SF-12), while the mental health status (MCS of the SF-12) remained unaffected [34]. We found no difference in QALY between IG and CG. There are two possible explanations. First, as QALY are based on HRQL, it could be the case that the changes in symptom severity might not have been strong enough to affect HRQL [40, 41]. Second, as we used the three level version of the EQ-5D to measure HRQL, it is possible to explain the absence of a difference in effects between IG and CG by the reduced responsiveness (sensitivity to change) of the EQ-5D-3L [71, 72]. An additional limitation resulting for the choice of the EQ-5D-3L is that this analysis is based on QALY. We are aware that there might have been other outcome parameters in this study that could have been used, like the PCS, the MCS or the PHQ-9. We did not consider these potential endpoints for two reasons. First, the pre-specified analytical concept determined QALY as endpoint of the analysis. Second, while there are commonly accepted willingness-to-pay thresholds for the ICER presented as cost per QALY, there are no thresholds for the ICER presented as cost per point of the PCS/MCS/PHQ-9. Next, we have to indicate a methodological limitation regarding our subgroup analyses. GP in the IG determined specific depression diagnoses based on the ICD-10 criteria and recommended an initial treatment based on the degree of severity of the ICD-10 diagnosis. As ICD-10 diagnoses were not determined in the CG, we were not able to use these diagnoses to classify patients into subgroups. Consequently, we used the PHQ-9 to categorize patients. This decision means that some patients were treated in a way that diverged from the way they were analysed. This does not affect the analysis of the complete sample, but could have led to a bias in our subgroup analyses that cannot be completely quantified. To get an idea of the potential of the bias, we compared the patients who were consistently diagnosed by both approaches to those who were categorized in different groups (data not shown). We found no noticeable differences in costs, especially healthcare costs. For example, in the group of patients with mild depression (consistent diagnoses made by the PHQ-9 and ICD-10) the cost were still higher than for those cases who were diagnosed as mild by the PHQ-9 and as moderate or severe based on the ICD-10 criteria. Additionally, the treatment costs for consistently diagnosed mildly depressed patients were still higher than those for the consistently moderately depressed patients. This noticeable finding appears to be stable to a certain degree.

Furthermore, we have to consider that the use of patient questionnaires is associated with a risk of missing values and recall bias. The degree of missing values was manageable and was handled by an elaborated approach [55–58]. The presence of a recall bias, which could have been unbalanced between the groups, cannot be ruled out or controlled. Additionally, in the interpretation of the results, we have to keep in mind that the randomization was not stratified for the subgroups. This means that the composition of the subgroups was not necessarily evenly allocated. For this reason, we adjusted the analyses in the subgroups for the group specific significant baseline differences.

## Conclusion

We found no evidence that our intervention is cost-effective over a one-year period. However, as there is evidence that guideline-based stepped care approaches for the treatment of depression can be cost-effective, we do not want to rule out that an adapted version of our intervention could be cost-effective. Consequently, there is further research needed to adapt our intervention and to develop implementation strategies that make cost-effective service delivery possible.

## Data Availability

Data are available from the corresponding author on reasonable request.

## Abbreviations

95%-CI: 95%-confidence interval
$\overline{\mathrm{C}}$: Mean total Costs
CEAC: Cost-Effectiveness Acceptability Curve
CG: Control Group
CSSRI: Client Sociodemographic and Service Receipt Inventory
$$ \overline{\mathrm{E}} $$: Mean Effects
GP: General Practitioner
HRQL: Health-related Quality of Life
ICER: Incremental Cost Effectiveness Ratio
IG: Intervention Group
MCS: Mental Component Score
MICE: Multiple Imputation using Chained Eqs
NB: Net Benefit
PCS: Physical Component Score
PHQ: Patient Health Questionnaire
QALY: Quality Adjusted Life Year
SCM: Stepped Care Model
SF-12: Short Form 12
TAU: Treatment As Usual
